# Osteogenic CpG Oligodeoxynucleotide, iSN40, Inhibits Osteoclastogenesis in a TLR9-Dependent Manner

**DOI:** 10.3390/life14121572

**Published:** 2024-11-30

**Authors:** Rena Ikeda, Chihaya Kimura, Yuma Nihashi, Koji Umezawa, Takeshi Shimosato, Tomohide Takaya

**Affiliations:** 1 Department of Agriculture, Graduate School of Science and Technology, Shinshu University, 8304 Minami-minowa, Kami-ina, Nagano 399-4598, Japan; 2 Department of Science and Technology, Graduate School of Medicine, Science and Technology, Shinshu University, 8304 Minami-minowa, Kami-ina, Nagano 399-4598, Japan; 3 Department of Agricultural and Life Sciences, Faculty of Agriculture, Shinshu University, 8304 Minami-minowa, Kami-ina, Nagano 399-4598, Japan; 4 Department of Biomolecular Innovation, Institute for Biomedical Sciences, Shinshu University, 8304 Minami-minowa, Kami-ina, Nagano 399-4598, Japan; 5 Institute for Aqua Regeneration, Shinshu University, 8304 Minami-minowa, Kami-ina, Nagano 399-4598, Japan

**Keywords:** CpG oligodeoxynucleotide (CpG-ODN), osteoclast, osteoclastogenesis, osteogenetic oligodeoxynucleotide (osteoDN), Toll-like receptor 9 (TLR9)

## Abstract

A CpG oligodeoxynucleotide (CpG-ODN), iSN40, was originally identified as promoting the mineralization and differentiation of osteoblasts, independent of Toll-like receptor 9 (TLR9). Since CpG ODNs are often recognized by TLR9 and inhibit osteoclastogenesis, this study investigated the TLR9 dependence and anti-osteoclastogenic effect of iSN40 to validate its potential as an osteoporosis drug. The murine monocyte/macrophage cell line RAW264.7 was treated with the receptor activator of nuclear factor-κB ligand (RANKL) to induce osteoclast differentiation, then the effect of iSN40 on was quantified by tartrate-resistant acid phosphatase (TRAP) staining and real-time RT-PCR. iSN40 completely inhibited RANKL-induced differentiation into TRAP^+^ multinucleated osteoclasts by suppressing osteoclastogenic genes and inducing anti-/non-osteoclastogenic genes. Treatment with a TLR9 inhibitor, E6446, or a mutation in the CpG motif of iSN40 abolished the intracellular uptake and anti-osteoclastogenic effect of iSN40. These results demonstrate that iSN40 is subcellularly internalized and is recognized by TLR9 via its CpG motif, modulates RANKL-dependent osteoclastogenic gene expression, and ultimately inhibits osteoclastogenesis. Finally, iSN40 was confirmed to inhibit the osteoclastogenesis of RAW264.7 cells cocultured with the murine osteoblast cell line MC3T3-E1, presenting a model of bone remodeling. This study demonstrates that iSN40, which exerts both pro-osteogenic and anti-osteoclastogenic effects, may be a promising nucleic acid drug for osteoporosis.

## 1. Introduction

Bone homeostasis is continuously maintained by bone remodeling, which is accomplished by balancing two biological processes. One is bone formation, in which osteoblasts express and mineralize the extracellular matrix to produce bone tissue, with differentiation into osteocytes that coordinate the functions of osteoblasts and osteoclasts. The other is bone resorption, in which monocytes differentiate as pre-osteoclasts into multinucleated osteoclasts that degrade the bone matrix [1]. Osteogenesis and osteoclastogenesis regulate each other to maintain their balance. Osteoblasts secrete the receptor activator of nuclear factor-κB ligand (RANKL), which is recognized by a RANKL receptor (RANK) on the pre-osteoclasts to promote osteoclast formation. Osteoblasts also produce a soluble RANKL decoy, osteoprotegerin (OPG), which interferes with RANKL signaling and inhibits osteoclastogenesis. In contrast, the RANK expressed by osteoclasts binds to RANKL in osteoblasts and enhances osteoblastic activity [1]. Toll-like receptors (TLRs) are involved in bone metabolism and remodeling. The activation of TLR2 and TLR4 in osteoblasts increases RANKL expression, depending on Myd88 without affecting the OPG level. The activation of TLR2, TLR4, and TLR9 in pre-osteoclasts impairs RANKL-induced osteoclastogenesis. However, in the absence of RANKL, TLR agonists are able to trigger osteoclast differentiation and extensive bone loss [2]. TLRs are one of the key receptors that sense the microenvironment and also modulate intercellular signaling between osteoblasts and osteoclasts to maintain bone homeostasis. Bone remodeling becomes functionally uncoupled with age, leading to bone mass loss and, eventually, osteoporosis [3]. In the elderly, several factors such as sclerostin, produced by osteocytes, increase bone resorption relative to bone formation [4,5]. Therefore, a humanized monoclonal anti-sclerostin antibody, romosozumab, which induces osteogenesis and inhibits osteoclastogenesis [6], has been used in osteoporosis therapy [7]. The success of romosozumab demonstrates that a dual effect on both osteoblasts and osteoclasts is beneficial in the treatment of osteoporosis. However, antibody drugs are still expensive and difficult to mass-produce for osteoporosis patients using the current technology.

Oligonucleotides have been studied and applied in clinical settings as next-generation drugs, due to their various advantages in chemical synthesis, variety of modifications, low-cost manufacturing, and storage stability [8]. For example, single-stranded oligodeoxynucleotides (ODNs) with unmethylated CpG motifs (CG sequences), which are typically generated from viral and bacterial genomes, often form palindromic structures to expose the CpG motifs on their molecular surface; these are recognized by TLR9 and initiate innate immune responses [8,9].Apart from that, we have reported that bacterial non-CpG-ODNs can become TLR9-independent aptamers that specifically interact with target molecules. For instance, iSN04 is an 18-base telomeric ODN derived from the lactic acid bacterium genome that forms a globular structure, binds to nucleolin, and regulates the fate of muscle lineage [10,11,12,13,14,15]. These viral and microbial genome-derived CpG-ODNs and aptamers are considered promising seeds for nucleic acid drugs that express bioactivities in various ways. The therapeutic potential of oligonucleotides has been anticipated, but only 21 nucleic acid drugs have been approved. Twelve of these are antisense oligonucleotides and six are small interfering RNAs; only one CpG-ODN and two aptamers have been commercialized (see the current update of a previous report [8]). Although CpG-ODNs as TLR9 ligands are being used in dozens of clinical trials for anti-cancer therapy by modulating the immune system [9], there are no ODNs being clinically tested for osteoporosis.

In the field of bone research, several osteogenetic ODNs (osteoDNs) have been identified that promote osteogenesis (Table 1). MT01, a non-CpG-ODN derived from the human mitochondrial genome, is the first osteoDN that induces osteogenic differentiation of the human osteoblast-like cell line MG63 [16,17], rat bone marrow mesenchymal stem cells [18], and the murine osteoblast cell line MC3T3-E1 [19]. Besides MT01, six designed CpG-ODNs (BW001, BW006, FC001, FC004, YW001, and YW002) were reported to promote the differentiation of MC3T3-E1 cells [19], but their TLR9 dependencies were not investigated. In contrast, CpG-2006 interferes with the osteoblastic differentiation of human mesenchymal stem cells in a TLR9-independent manner [20].

Furthermore, CpG-ODNs are known to inhibit RANKL-induced osteoclastogenesis in a TLR9-dependent manner. CpG-1826 blocks the osteoclast differentiation of murine bone marrow macrophages (mBMM) [23,26,27] but not that of human peripheral blood monocytes (hPBMC) [23]. Conversely, CpG-2006 works on hPBMC but not on mBMM. CpG-KSK13 was, therefore, designed to inhibit osteoclastogenesis in both mBMM and hPBMC [23]. CpG-1585 prevents osteoclast formation of the murine monocyte/macrophage cell line RAW264.7 by upregulating A20 deubiquitinase [22]. Similarly, YW001, YW002, and FC004, which were originally identified as CpG-osteoDNs [19], inhibit the osteoclastic differentiation of RAW264.7 cells, whereas a non-CpG-osteoDN, MT01, does not affect osteoclastogenesis [28]. The TLR9 dependencies of the anti-osteoclastogenic effects of these osteoDNs have not been clarified.

These studies demonstrated that elucidating their TLR9 dependencies of CpG-ODNs, which regulate the differentiation of osteoblasts and osteoclasts, may provide the medical seeds for osteoporosis therapy. We have recently identified iSN40, a novel CpG-osteoDN derived from the lactic acid bacterium genome, which strongly promotes the mineralization and differentiation of MC3T3-E1 osteoblasts in a TLR9-independent manner [21]. To establish iSN40 as an anti-osteoporotic molecule, this study investigated the anti-osteoclastogenic effect and TLR9 dependency of iSN40 on RAW264.7 cells.

## 2. Materials and Methods

### 2.1. Chemicals

The ODNs used in this study (Table 1), in which all phosphodiester bonds were phosphorothioated (PS) to enhance nuclease resistance, were synthesized and HPLC-purified (GeneDesign, Osaka, Japan). And, 6-FAM-ODNs are PS-ODNs that are conjugated to 6-carboxyfluorecein at their 5′ ends (GeneDesign). All ODNs were dissolved in endotoxin-free water. Recombinant murine RANKL (ab129136; Abcam, Cambridge, UK) was dissolved in phosphate-buffered saline (PBS). A TLR9 inhibitor, dihydrochloride (E6446) (MedChem Express, Monmouth Junction, NJ, USA), was dissolved in dimethyl sulfoxide. Ascorbic acid (Fujifilm Wako Chemicals, Osaka, Japan) was dissolved in endotoxin-free water. Equal volumes of solvents were used as the negative controls.

### 2.2. Culture and Osteoclastogenesis of RAW264.7 Cells

All cells were cultured at 37 °C with 5% CO_2_ throughout the experiments. RAW264.7 cells (91062702; Public Health England, London, UK) were maintained in DMEM (Nacalai, Osaka, Japan), supplemented with 10% fetal bovine serum (FBS) (HyClone; GE Healthcare, Salt Lake City, UT, USA) and a mixture of 100 units/mL penicillin and 100 μg/mL streptomycin (P/S) (Nacalai). For osteoclastogenesis, RAW264.7 cells were seeded on fresh dishes or plates, and 30 ng/mL of RANKL was added the next day (defined as day 0). The ODNs were simultaneously treated with RANKL. The ODN concentration in each experiment is described in the figure legends. To inhibit TLR9 function, 1 μM of E6446 was administered 3 h before ODN treatment. The medium containing RANKL, ODN, and E6446 was replaced every 2–3 days [29].

### 2.3. Tartrate-Resistant Acid Phosphatase (TRAP) Staining

RAW264.7 cells were seeded on 24-well plates (1.0 × 10^4^ cells/well) and induced osteoclastogenesis in the RANKL-containing medium for 5–7 days. The TRAP enzymatic activity of the differentiated osteoclasts was visualized using a TRAP Staining Kit (Fujifilm Wako Chemicals) according to the manufacturer’s instructions. Cell nuclei were stained with 1% methylene blue in 3% acetic acid. Bright-field images were captured using an EVOS FL Auto microscope (AMAFD1000; Thermo Fisher Scientific, Waltham, MA, USA). The number of nuclei in the TRAP^+^ multinucleated osteoclasts (with >3 nuclei) per field was counted using ImageJ software, version 1.52u (National Institute of Health, Bethesda, MD, USA) [30].

### 2.4. Bone Matrix Resorption Assay

RAW264.7 cells were seeded on calcium phosphate-coated 24-well plates (PG Research, Tokyo, Japan) (3.0 × 10^4^ cells/well) and induced osteoclastogenesis in RANKL-containing medium for 6 days. The cells were then lysed with 5% sodium hypochlorite. Phase-contrast images of the pits generated by the resorption of the calcium phosphate matrix by functional mature osteoclasts were captured using an EVOS FL Auto microscope. The pit area was measured using ImageJ software, version 1.52u.

### 2.5. Quantitative Real-Time RT-PCR (qPCR)

RAW264.7 cells were seeded on 30-mm (2.0 × 10^4^ cells/dish) or 60-mm (1.0 × 10^5^ cells/dish) dishes and induced osteoclastogenesis for 1–5 days. Total RNA from the cells was isolated using NucleoSpin RNA Plus (Macherey-Nagel, Düren, Germany) and was reverse-transcribed using ReverTra Ace qPCR RT Master Mix (TOYOBO, Osaka, Japan). qPCR was performed using the GoTaq qPCR Master Mix (Promega, Madison, WI, USA) with the StepOne Real-Time PCR System (Thermo Fisher Scientific). The amount of each transcript was normalized to that of the tyrosine 3-monooxygenase/tryptophan 5-monooxygenase activation protein zeta (*Ywhaz*) gene. Results are expressed as fold-changes. Primer sequences are listed in Table 2.

### 2.6. ODN Incorporation Assay

RAW264.7 cells were seeded on 8-well glass chamber slides (3000 cells/well) (SCS-N08; Matsunami Glass, Osaka, Japan) and treated with 5 μg/mL (0.79 μM) 6-FAM-iSN40 or 6-FAM-iSN40-GC for 30–60 min the next day. The cells were then washed with PBS, fixed with 2% paraformaldehyde, and stained with DAPI (Nacalai). Fluorescence images were captured using an EVOS FL Auto microscope with a 40× objective lens [10,11,15].

### 2.7. Multicanonical Molecular Dynamics (McMD) Simulation

The conformations of iSN40, iSN40-GC, and iSN41 were simulated using the trivial-trajectory parallelization (TTP)-McMD method [35], as described previously [10,21,36]. Briefly, the initial structure of an ODN molecule was built as a DNA helix model by NAB in AmberTools [37], and the force field of Amber ff99bsc0 was applied [38]. TTP-McMD was conducted to sample the equilibrated conformations at 310 K in the implicit solvent of water (igb = 1). The energy range of the multicanonical ensemble covered 270 K to 400 K. Sixty trajectories were used and five iterative runs were performed to achieve a flat distribution along the energy range. The production run was conducted for 100 ns in each trajectory (total 6 μs). Snapshots were saved every 200 ps. A total of 30,000 snapshots were sampled, and the 7033, 4039, and 5823 conformations of iSN40, iSN40-GC, and iSN41 at 310 K were obtained by the reweighting method. The representative ODN structures were taken from the centroid of structural clustering using AmberTools22 [39]. Structure images were generated using UCSF Chimera [40].

### 2.8. Principal Component Analysis (PCA)

The molecular structures of iSN40, iSN40-GC, and iSN41 obtained by TTP-McMD were subjected to PCA. All sampled conformations of the three ODNs were used to construct principal components (PCs). The sines and cosines of 122 dihedral angles in the DNA-backbone conformation were used as the elements of the feature vector per conformation. The vector contained 244 dimensions. Variance-covariance matrices (244 × 244 dimensions) were calculated from the vectors of the sampled conformations. The matrices were diagonalized, and the eigenvalues and eigenvectors were obtained. The projection of the largest eigenvalue onto the eigenvector corresponded to the first and second components of the PCA (PC1 and PC2). These calculations were performed using Python scripts [14].

### 2.9. Coculture of RAW264.7 and MC3T3-E1 Cells

Murine MC3T3-E1 cells (RCB1126; RIKEN BioResource Research Center, Tsukuba, Japan), maintained in a coculture medium consisting of α-MEM (Nacalai) supplemented with 10% FBS and P/S, were used as osteoblasts for coculture because iSN40 has been reported to promote their mineralization and differentiation [21]. Murine RAW264.7 cells were used as pre-osteoclasts for coculture to ensure intercellular communication with the MC3T3-E1 cells. For the coculture, RAW264.7 cells (3.0 × 10^4^ cells/well) and MC3T3-E1 cells (1.0 × 10^4^ cells/well) were seeded simultaneously in coculture medium on 24-well plates [41,42]. The next day (defined as day 0), the cells were treated with 100 ng/mL RANKL, 50 μg/mL ascorbic acid, and ODNs to induce osteoclastogenesis.

### 2.10. Statistical Analysis

Results are presented as the mean ± standard error. Statistical comparison among multiple groups was performed using the Tukey–Kramer test after a one-way analysis of variance. Statistical significance was set at *p* < 0.05.

## 3. Results

### 3.1. iSN40 Inhibits RANKL-Induced Osteoclastogenesis of RAW264.7 Cells

**Figure 1:**
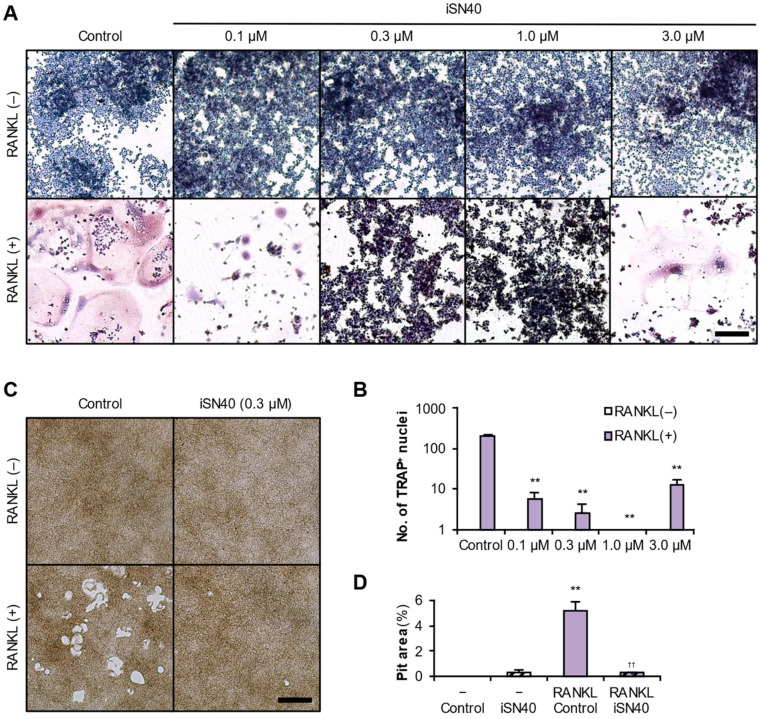
iSN40 inhibits the RANKL-induced osteoclastogenesis of RAW264.7 cells. (**A**) Representative images of the TRAP staining of RAW264.7 cells treated with 30 ng/mL RANKL and 0.1–3.0 μM iSN40 for 7 days. Scale bar, 100 μm. (**B**) The number of nuclei in the TRAP^+^ osteoclasts was quantified. No TRAP^+^ cells were observed in any of the RANKL(−) groups. ** *p* < 0.01 vs. RANKL(+)/control. *n* = 4 fields. (**C**) Representative images of the pits generated in the bone matrix resorption assay of RAW264.7 cells treated with 100 ng/mL RANKL and 0.3 μM iSN40 for 6 days. Scale bar, 100 μm. (**D**) The pit area was quantified. ** *p* < 0.01 vs. RANKL(−)/control; ^††^ *p* < 0.01 vs. RANKL(+)/control. *n* = 3 fields.

RAW264.7 cells were induced osteoclastogenic differentiation using RANKL, treated with iSN40, and then stained for TRAP enzymatic activity, a marker of mature osteoclasts. As shown in Figure 1A, on day 7 after differentiation, multinucleated TRAP^+^ osteoclasts were formed in the RANKL-treated control group, but their osteoclastogenesis was disrupted by iSN40. The effect of iSN40 was concentration-dependent. Osteoclast formation was significantly but incompletely suppressed by iSN40 at 0.1 μM, absolutely inhibited at 0.3 and 1.0 μM, and partially downregulated at 3.0 μM (Figure 1B, displayed logarithmically). The results indicated that iSN40 inhibits the RANKL-induced osteoclastogenesis of RAW264.7 cells at optimal doses of 0.3–1.0 μM, a concentration range that overlaps with those of other anti-osteoclastogenic CpG-ODNs [22,23,25,26,27,28]. In the following experiments, RAW264.7 cells were treated with 0.3 μM ODNs, unless otherwise noted.

Next, RAW264.7 cells were induced to form osteoclasts on calcium phosphate-coated plates and their bone resorption capacity was assessed. After osteoclastogenesis, the pits generated by resorption of the calcium phosphate matrix by functional mature osteoclasts were observed and quantified. As shown in Figure 1C,D, the pit area was significantly increased in the RANKL-treated group, but the RANKL-dependent pit formation was completely inhibited by iSN40. This result indicates that iSN40 strongly interferes with RANKL-induced functional osteoclastogenesis and may prevent bone resorption.

**Figure 2:**
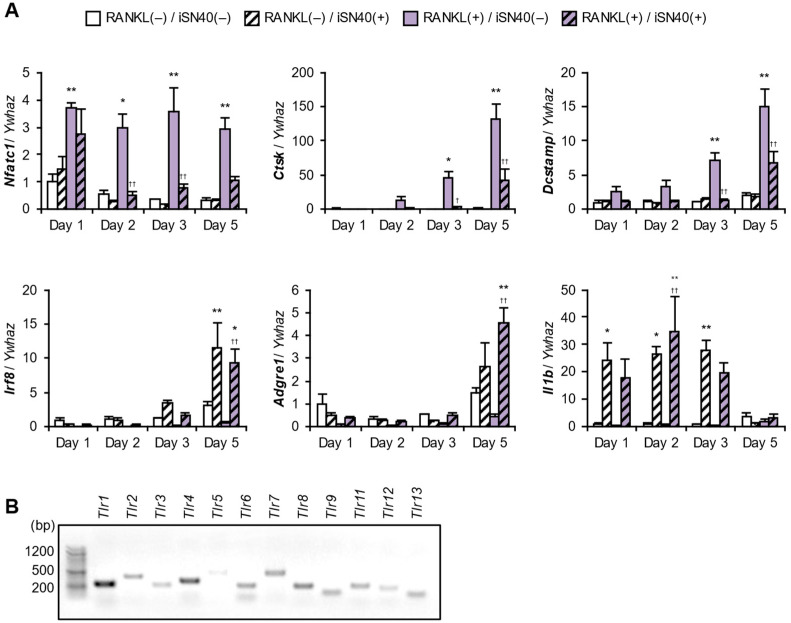
iSN40-dependent gene expression changes in RAW264.7 cells. (**A**) qPCR results of RAW264.7 cells treated with 30 ng/mL RANKL and 0.3 μM iSN40. * *p* < 0.05, ** *p* < 0.01 vs. RANKL(−)/iSN40(−); ^†^ *p* < 0.05, ^††^ *p* < 0.01 vs. RANKL(+)/iSN40(−) on each day. *n* = 3. (**B**) Total RNA from RAW264.7 cells was subjected to RT-PCR (40 cycles), then the PCR products of TLR genes were subjected to 1.5% agarose gel electrophoresis and stained with ethidium bromide.

qPCR revealed the iSN40-dependent gene expression changes in RAW264.7 cells (Figure 2A). iSN40 significantly downregulated RANKL-induced osteoclastogenic gene expression; nuclear factor of activated T cell 1 (*Nfatc1*) is the RANKL-responsive transcription factor that initiates osteoclast differentiation [11], cathepsin K (*Ctsk*) is an osteoclast-specific cysteine protease for bone resorption, and the dendrocyte-expressed seven transmembrane protein (*Dcstamp*) is essential for cell fusion to form multinucleated osteoclasts. In contrast, iSN40 significantly upregulated anti-osteoclastogenic transcription, even in the presence of RANKL; interferon regulatory factor 8 (IRF-8) (*Irf8*) is the competing transcription factor for NFATc1, which enhances macrophagic and suppresses osteoclastogenic gene expression [2,43]. Consistent with this finding, iSN40 strikingly induced a macrophage marker, adhesion G protein-coupled receptor E1 (*Adgre1*; F4/80), despite RANKL treatment. iSN40 significantly increased interleukin 1β (IL-1β) (*Il1b*) mRNA levels independently of RANKL. Since RAW264.7 cells express all TLR genes (Figure 2B), and TLR ligands arrest osteoclast differentiation [2], it was hypothesized that iSN40 serves as a CpG-ODN recognized by TLR9 to induce immune responses and maintain the cells in a macrophage lineage.

### 3.2. iSN40 Inhibits Osteoclastogenesis in a TLR9-Dependent Manner

**Figure 3:**
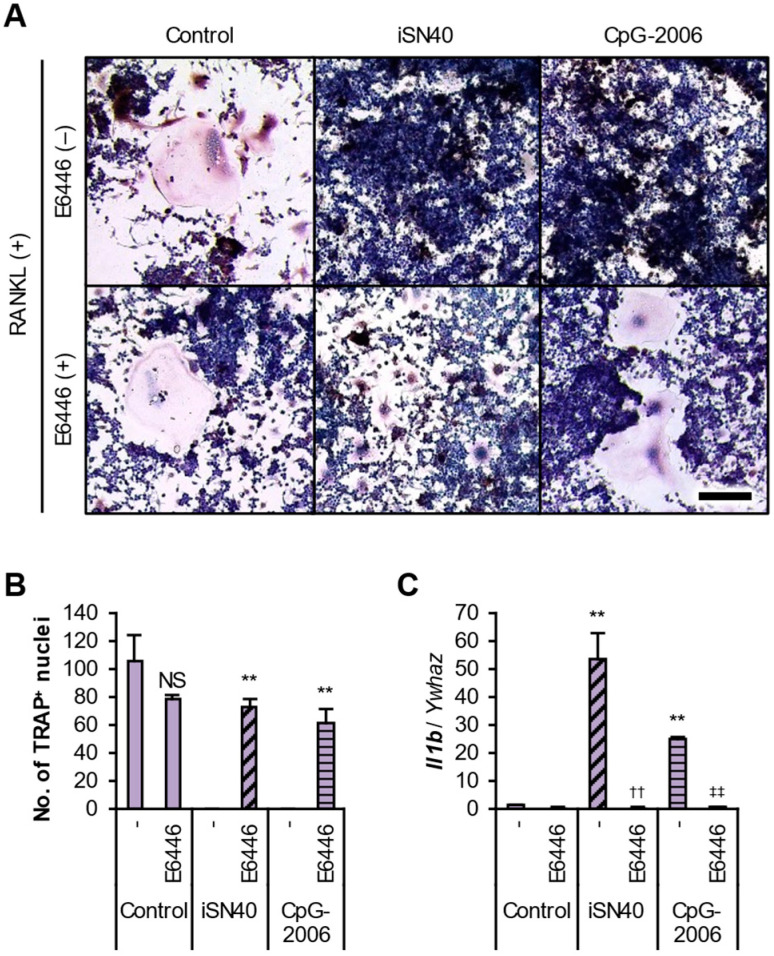
iSN40 inhibits osteoclastogenesis in a TLR9-dependent manner. (**A**) Representative images of the TRAP staining of RAW264.7 cells treated with 30 ng/mL RANKL, 0.3 μM ODN, and 1 μM E6446 for 6 days. Scale bar, 100 μm. (**B**) The number of nuclei in TRAP^+^ osteoclasts was quantified. No TRAP^+^ cells were observed in the iSN40/E6446(−) and CpG-2006/E6446(−) groups. NS, no significant difference, ** *p* < 0.01 vs. each E6446(−) group. *n* = 4 fields. (**C**) qPCR results of RAW264.7 cells treated with 30 ng/mL RANKL, 0.3 μM ODNs, and 1 μM E6446 for 24 h. ** *p* < 0.01 vs. control/E6446(−), ^††^ *p* < 0.01 vs. iSN40/E6446(−), ^‡‡^ *p* < 0.01 vs. CpG-2006/E6446(−). *n* = 3.

The TLR9 dependency of iSN40 was investigated using a TLR9 inhibitor, E6446, and a known CpG-ODN, CpG-2006, which has been reported to activate TLR9 in RAW264.7 cells [24]. As shown in Figure 3A, CpG-2006 completely inhibited the RANKL-induced osteoclastogenesis of RAW264.7 cells to the same extent as iSN40. As expected, pre-treatment with E6446 reversed the anti-osteoclastogenic effects of iSN40 and CpG-2006. Although the TRAP^+^ cells were still small in the iSN40 and CpG-2006 groups with E6446, the reduction in the number of nuclei in TRAP^+^ cells by iSN40 or CpG-2006 was fully recovered by E6446 (Figure 3B). qPCR also showed that E6446 completely blocked iSN40- and CpG-2006-induced *Il1b* expression (Figure 3C). These data demonstrate that the recognition of iSN40 by TLR9 is an essential step in the interruption of RANKL-induced osteoclast differentiation.

### 3.3. The CpG Motif Within iSN40 Is Essential for Its Anti-Osteoclastogenic Effect

**Figure 4:**
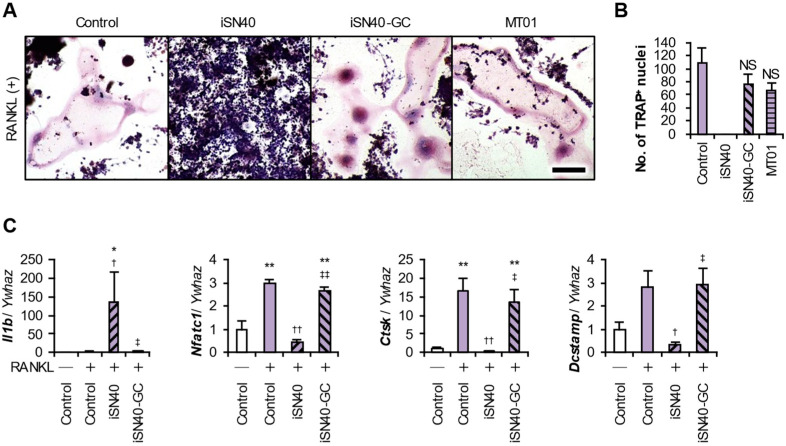
The CpG motif is essential for the anti-osteoclastogenic effect of iSN40. (**A**) Representative images of the TRAP staining of RAW264.7 cells treated with 30 ng/mL RANKL and 0.3 μM ODN for 7 days. Scale bar, 100 μm. (**B**) The number of nuclei in the TRAP^+^ osteoclasts was quantified. No TRAP^+^ cells were observed in the iSN40 group. NS, no significant difference vs. control. *n* = 4 fields. (**C**) qPCR results of RAW264.7 cells treated with 30 ng/mL RANKL, 0.3 μM ODNs for 24 h (*Il1b*) or 5 days (*Nfatc1*, *Ctsk*, and *Dcstamp*). * *p* < 0.05, ** *p* < 0.01 vs. RANKL(−)/control; ^†^ *p* < 0.05, ^††^ *p* < 0.01 vs. RANKL(+)/control; ^‡^ *p* < 0.05, ^‡‡^ *p* < 0.01 vs. RANKL(+)/iSN40. *n* = 3.

TLR9 recognizes the unmethylated CpG di-deoxynucleotide motifs within CpG-ODNs [44]. To investigate the impact of the CpG motif within iSN40 (5′-GGA ACG ATC CTC AAG CTT-3′) on osteoclastogenesis, iSN40-GC (5′-GGA AGC ATC CTC AAG CTT), in which the CG sequence was substituted with GC (underlined sequences), was used. iSN40-GC did not inhibit the RANKL-induced osteoclast formation of RAW264.7 cells at all (Figure 4A,B). Importantly, MT01, another osteoDN having no CpG motif, also showed no action on osteoclastogenesis, demonstrating that the pro-osteogenic and anti-osteoclastogenic effects of osteoDNs are independent and can be separated. qPCR reproducibly showed that iSN40-GC did not initiate immune responses such as *Il1b* expression and did not downregulate the expression of osteoclastogenic genes including *Nfatc1*, *Ctsk*, and *Dcstamp* (Figure 4C). These data clearly indicate that the CpG motif is essential for the anti-osteoclastogenic effect of iSN40 via its recognition by TLR9.

### 3.4. The CpG Motif of iSN40 Is Important for Intracellular Incorporation

**Figure 5:**
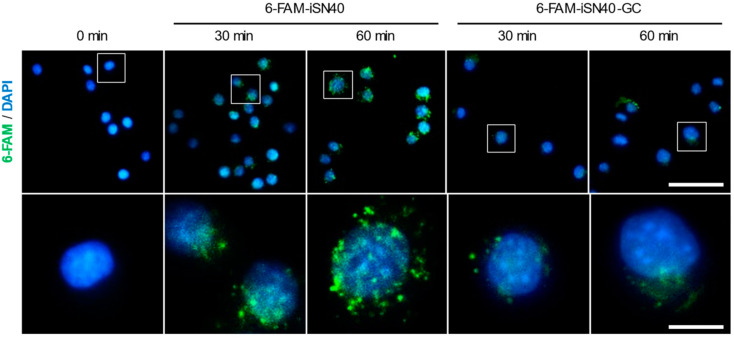
Intracellular incorporation of iSN40. Representative fluorescence images of RAW264.7 cells treated with 5 μg/mL 6-FAM-iSN40 or 6-FAM-iSN40-GC for 30–60 min. The bottom panels are magnifications of rectangular areas within the top panels. Scale bars, 50 μm (**top panels**) and 10 μm (**bottom panels**).

CpG-ODNs must be taken up intracellularly to associate with TLR9 in the endosomes [45]. A previous study has demonstrated that both iSN40 and iSN40-GC were similarly incorporated into the cytoplasm of MC3T3-E1 cells, which do not express TLR9, within 30 min without a carrier [21]. As shown in Figure 5, 6-FAM-iSN40, when administered to RAW264.7 cells, was autonomously internalized into the cytoplasm within 30 min and localized in the endosome-like vesicles within 60 min, while 6-FAM-iSN40-GC was less fully incorporated compared to iSN40, and the vesicles were not clearly observed. These results suggest that the CpG motif of iSN40 is important for its intracellular incorporation and subcellular localization, probably to TLR9 in the endosomes, which is related to the anti-osteoclastogenic effect.

### 3.5. The Location of the CpG Motif Is Essential for iSN40 Activity

**Figure 6:**
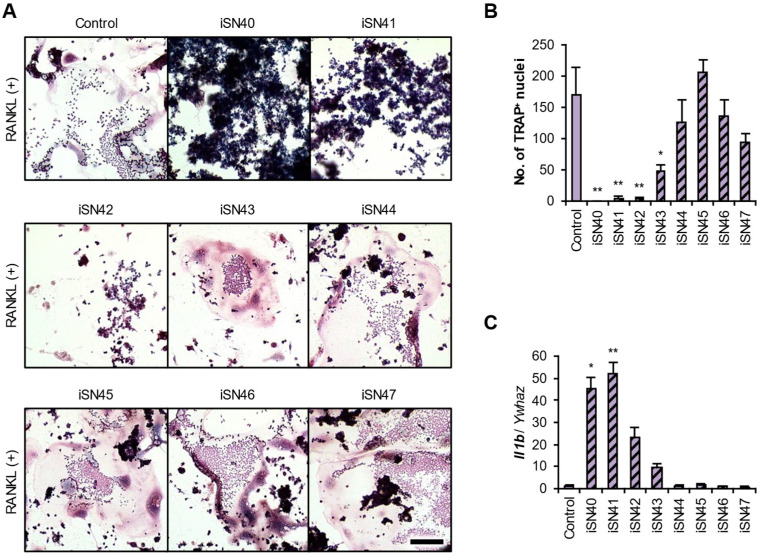
Anti-osteoclastogenic effects of the iSN40 variants. (**A**) Representative images of the TRAP staining of RAW264.7 cells treated with 30 ng/mL RANKL and 0.3 μM ODN for 7 days. Scale bar, 100 μm. (**B**) The number of nuclei in the TRAP^+^ osteoclasts was quantified. No TRAP^+^ cells were observed in the iSN40 group. * *p* < 0.05, ** *p* < 0.01 vs. control. *n* = 4 fields. (**C**) qPCR results of RAW264.7 cells treated with 30 ng/mL RANKL and 0.3 μM ODN for 24 h. * *p* < 0.05, ** *p* < 0.01 vs. control. *n* = 3.

It is not necessarily the case that all CpG-ODNs can be TLR9 ligands [44]. The iSN40 variants, iSN41–iSN47 (Table 1), have a CpG motif (except for iSN45, which has no CpG motif as a negative control) but are not osteoDNs [21]. The anti-osteoclastogenic effects and immune responses of these ODNs were investigated. iSN41 and iSN42 completely inhibited and iSN43 partially inhibited osteoclastogenesis, but the others showed no significant effects (Figure 6A,B). Correspondingly, iSN41 markedly induced *Il1b* expression as well as iSN40 (Figure 6C). iSN41, the variant most homologous to iSN40, showed almost the same bioactivities as iSN40 in RAW264.7 cells. Overall, iSN40–iSN43, with a CpG motif in their 5′ regions (5′-CpG), exhibited anti-osteoclastogenic and immunostimulatory effects, whereas iSN46 and iSN47, with a 3′-CpG dinucleotide, did not. iSN44, with a CpG motif at its 5′ terminus, and iSN45, with no motif, also showed no activities. These results are consistent with those of a previous study, which reported that CpG-ODNs with a 5′-CpG are more active than those with a 3′-CpG [46]. These suggest that the CpG motif located at the 5–6th residues is essentially required for the bioactivities of iSN40.

### 3.6. Molecular Simulation of the iSN40 Structure

**Figure 7:**
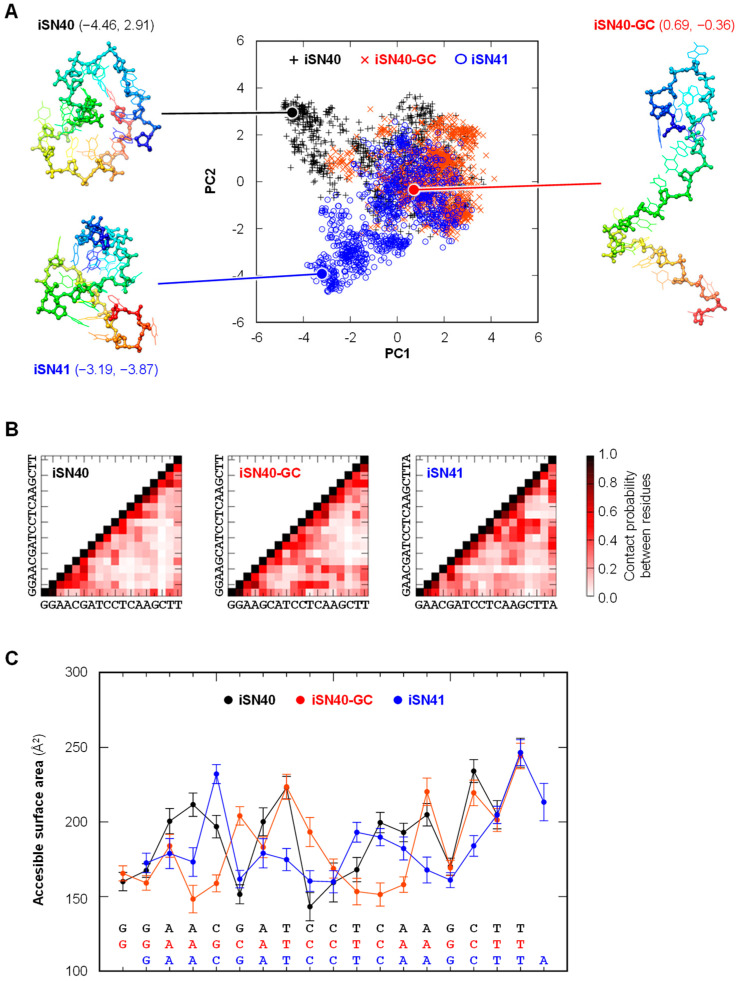
Molecular simulation of the structures of iSN40, iSN40-GC, and iSN41. (**A**) The structures of iSN40, iSN40-GC, and iSN41, simulated by TTP-McMD, are shown in a two-dimensional plot on the PCA space reconstructed by PC1 (contribution, 0.083) and PC2 (0.064). The molecular structures, shown as a rainbow-colored stick model from the 5′ end (blue) to the 3′ end (red), are representative conformations taken from the centroid of the structural clustering. The PCA coordinate (PC1, PC2) of each conformation is indicated. (**B**) The contact probabilities between residues within iSN40, iSN40-GC, and iSN41. (**C**) The accessible surface areas of the residues of iSN40, iSN40-GC, and iSN41.

iSN40 and iSN41 exerted anti-osteoclastogenic effects in a TLR9-dependent manner, although a non-CpG-ODN, iSN40-GC, did not. In contrast, iSN40 and iSN40-GC can promote osteogenesis independently of TLR9, but iSN41 does not do so [21]. These results suggest that the pro-osteogenic and anti-osteoclastogenic effects of iSN40 are based on different mechanisms and can be functionally separated. To analyze the relationship between the molecular structures and functions of iSN40, iSN40-GC, and iSN41, their conformations were computationally simulated using TTP-McMD. The resulting structures were subjected to PCA analysis and clustered to abstract their representative conformations (Figure 7A). The molecular structures of iSN40, iSN40-GC, and iSN41 partially overlapped, probably because their sequences were nearly identical. However, detailed analysis revealed sub-molecular differences. As shown in Figure 7B, the contact probabilities (analogous to distance) between the residues within iSN40 were similar to those of iSN40-GC. In particular, the 3′ terminal sequences, AGCTT, were structurally open (residues not in contact with each other) in iSN40 and iSN40-GC, whereas in iSN41, the 15–16th CT interacted with the 9–10th CT. Also, the accessible surface area (analogous to exposure) patterns of AGCTT closely resembled each other in iSN40 and iSN40-GC, but not in iSN41 (Figure 7C). These data suggest that the common AGCTT properties of iSN40 and iSN40-GC may contribute to their TLR-independent pro-osteogenic effects. Focusing on the CpG motifs of iSN40 and iSN41, the 6th G was in close contact with the 4–5th AC in iSN40 (contact between the 5th G and the 3–4th AC in iSN41), suggesting that this is the basis of their TLR9-dependent anti-osteoclastogenic actions.

### 3.7. iSN40 Exerts an Anti-Osteoclastogenic Effect in the Presence of Osteoblasts

**Figure 8:**
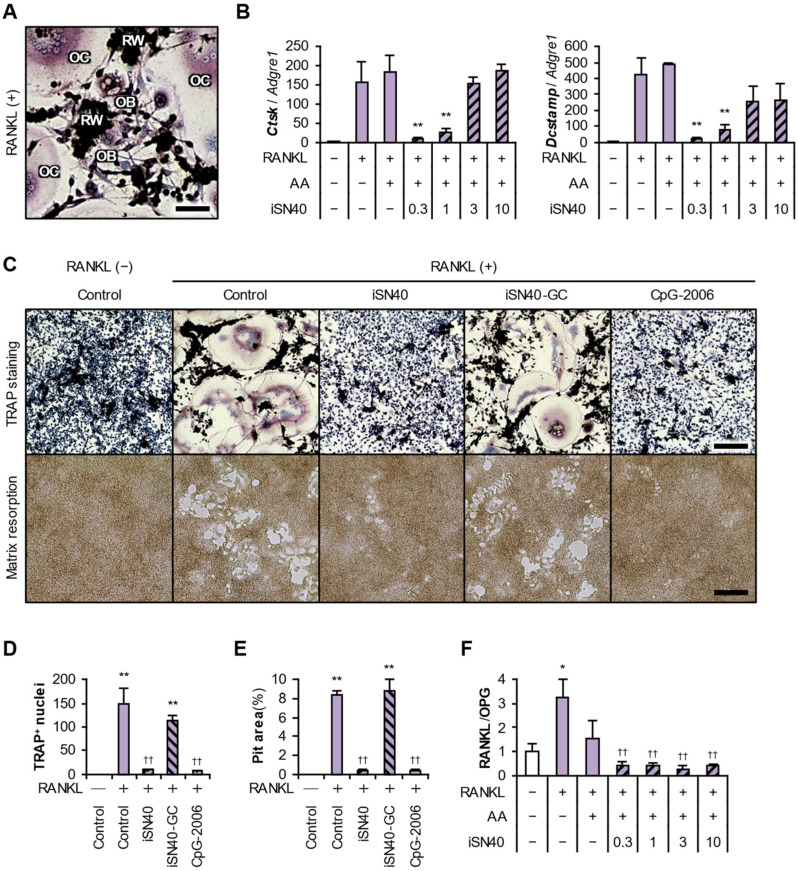
Anti-osteoclastogenic effect of iSN40 in the coculture of RAW264.7 and MC3T3-E1 cells. (**A**) Representative images of the TRAP staining of cocultured RAW264.7 and MC3T3-E1 cells treated with 100 ng/mL RANKL for 4 days. RW, undifferentiated RAW264.7 cells (dense nuclei in black); OC, differentiated TRAP^+^ multinucleated osteoclasts; OB, fibroblast-like MC3T3-E1 osteoblasts. Scale bar, 25 μm. (**B**) qPCR results of cocultured RAW264.7 and MC3T3-E1 cells treated with 100 ng/mL RANKL, 50 μg/mL ascorbic acid (AA), and 0.3–10 μM iSN40 for 4 days. ** *p* < 0.01 vs. RANKL(+)/AA(+)/iSN40(−). *n* = 3. (**C**) Representative images of the TRAP staining of cocultured RAW264.7 and MC3T3-E1 cells treated with 100 ng/mL RANKL and 0.3 μM ODN for 4 days (top panels). Representative images of the pits generated in the bone matrix resorption assay of cocultured RAW264.7 and MC3T3-E1 cells treated with 100 ng/mL RANKL and 0.3 μM ODN for 6 days (bottom panels). Scale bars, 100 μm. (**D**) The number of nuclei in TRAP^+^ osteoclasts in panel C was quantified. ** *p* < 0.01 vs. RANKL(−)/control, ^††^ *p* < 0.01 vs. RANKL(+)/control. *n* = 3 fields. (**E**) Scale bar, 100 μm. (**E**) The pit area in panel C was quantified. ** *p* < 0.01 vs. RANKL(−)/control, ^††^ *p* < 0.01 vs. RANKL(+)/control. *n* = 3 fields. (**F**) qPCR results of the ratio of RANKL (*Tnfsf11*) to OPG (*Tnfrsf11b*) of the same samples in panel B. * *p* < 0.05 vs. RANKL(−)/AA(−)/iSN40(−), ^††^ *p* < 0.01 vs. RANKL(+)/AA(−)/iSN40(−). *n* = 3.

Finally, the anti-osteoclastogenic effect of iSN40 was tested in the presence of osteoblasts by coculturing RAW264.7 cells and MC3T3-E1 osteoblasts [41,42], because osteoclasts and osteoblasts mutually regulate their differentiation through RANKL–RANK signaling to maintain the balance between bone resorption and formation in vivo [1]. Since both iSN40 and iSN40-GC promote the mineralization and differentiation of osteoblasts independently of TLR9 [21], their pro-osteogenic effects may indirectly influence the osteoclastogenesis of RAW264.7 cells in coculture with MC3T3-E1 cells. Confirmation of the anti-osteoclastogenic effect of iSN40 in the presence of osteoblasts is, therefore, important for animal studies and clinical applications. The cocultured RAW264.7 and MC3T3-E1 cells were subjected to RANKL-induced osteoclastogenesis for 4 days. As shown in Figure 8A, the undifferentiated RAW264.7 cells, differentiated TRAP^+^ multinucleated osteoclasts, and fibroblast-like MC3T3-E1 osteoblasts were in close contact with each other, allowing paracrine signaling among them during differentiation in this system.

Ascorbic acid has been widely used, not only to induce differentiation of MC3T3-E1 cells [47], but is also known to inhibit RANKL-induced osteoclastogenesis of RAW264.7 cells at higher doses [48], which is promising as a potential nutritional vitamin for osteoporosis therapy [49]. The RANKL-induced expression levels of the osteoclastogenic genes, *Ctsk* and *Dcstamp*, were quantified in the coculture condition treated with RANKL and 50 μg/mL ascorbic acid. Transcripts were normalized to that of *Adgre1*, which is expressed only in RAW264.7 cells, not in MC3T3-E1 cells. As shown in Figure 8B, ascorbic acid did not alter the RANKL-induced *Ctsk* and *Dcstamp* levels in this system. iSN40 significantly inhibited osteoclastogenesis at a dose of 0.3–1.0 μM but not at 3–10 μM, as observed in the single RAW264.7 cell culture (Figure 1A,B). TRAP staining clearly visualized the finding that iSN40 and CpG-2006, but not iSN40-GC, inhibited the RANKL-induced osteoclastogenesis in the coculture system (Figure 8C,D). The bone matrix resorption assays also showed that iSN40 and CpG-2006 significantly interfered with RANKL-induced pit formation by functional osteoclasts (Figure 8C,E). These results demonstrate that iSN40 can exert its anti-osteoclastogenic effect, based on its CpG motif and TLR9, even in the presence of osteoblasts.

Furthermore, the effect of iSN40 on osteoblasts in this coculture system was investigated. Osteoblasts produce RANKL and OPG (an inhibitory decoy of RANKL) [1], and the RANKL/OPG ratio reflects the physiological balance of bone formation and resorption. Clinically speaking, the serum RANKL/OPG ratio has been suggested to be associated with the risk of osteoporotic fracture [50]. As shown in Figure 8F, qPCR revealed that the mRNA level ratio of RANKL (*Tnfsf11*) to OPG (*Tnfrsf11b*) was significantly increased by RANKL treatment, but markedly decreased by cotreatment with ascorbic acid and iSN40. This result demonstrates that iSN40 affects MC3T3-E1 osteoblasts, altering the expression levels of RANKL and OPG, even in the presence of cocultured RAW264.7 cells. It strongly suggests that iSN40 will be able to exert both a pro-osteogenic effect on osteoblasts and an anti-osteoclastogenic effect on osteoclasts during bone remodeling.

**Figure 9:**
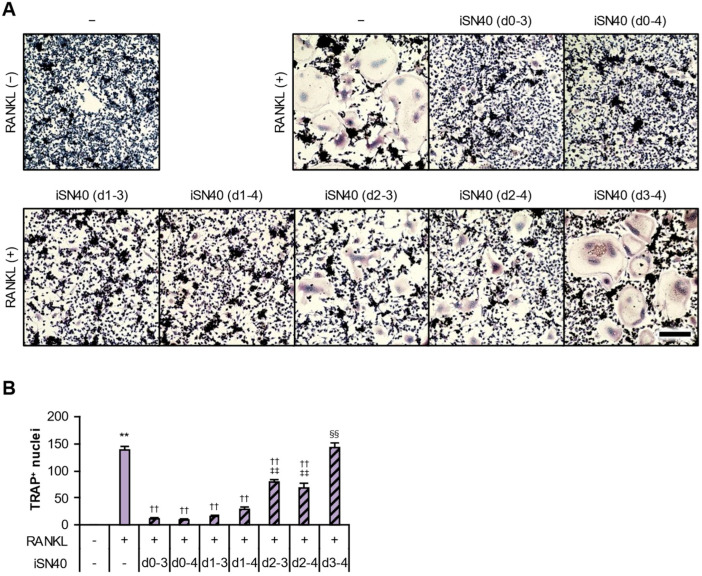
Time course of anti-osteoclastogenic effect of iSN40. (**A**) Representative images of TRAP staining of cocultured RAW264.7 and MC3T3-E1 cells treated with 100 ng/mL RANKL for 4 days and 0.3 μM iSN40 for defined periods. d, differentiation days with iSN40 treatment. Scale bar, 100 μm. (**B**) The number of nuclei in TRAP^+^ osteoclasts was quantified. ** *p* < 0.01 vs. control/RANKL(−); ^††^ *p* < 0.01 vs. control/RANKL(+); ^‡‡^ *p* < 0.01 vs. d0–3, d0–4, d1–3, and d1–4; ^§§^ *p* < 0.01 vs. d2–3 and d2–4. *n* = 3 fields.

In the above coculture experiments, iSN40 was treated throughout the 4–6 days of RANKL-induced differentiation. To determine the effective period of anti-osteoclastogenic treatment, iSN40 was administered and removed at different time points. As shown in Figure 9A,B, iSN40 treatment during differentiation days 1 to 3 (d1–3) was sufficient to inhibit osteoclast formation, compared to the conventional full treatment (d0–4), while iSN40 administration from day 2 (d2–3 and d2–4) partially allowed osteoclastogenesis, and that from day 3 (d3–4) showed no inhibitory effect. These data suggest that the first 2 days of osteoclast differentiation are essential for the anti-osteoclastogenic mechanism of iSN40. This finding corresponds well with previous studies reporting that the treatment of pre-osteoclasts with RANKL and TLR agonists in the early stages of differentiation arrests osteoclastogenesis [2].

## 4. Discussion

This study demonstrated that a CpG-osteoDN, iSN40, completely inhibits the RANKL-induced osteoclastic differentiation of RAW264.7 cells. The anti-osteoclastogenic effect of iSN40 was abolished by a TLR9 inhibitor or by the substitution of its CpG motif with a GC sequence. These results clearly indicate that the CpG motif of iSN40 is recognized by TLR9 in RAW264.7 cells as interfering with osteoclast differentiation. The optimal dose of iSN40 was 0.3–1.0 μM, which corresponds to that of known CpG-ODNs for TLR9-dependent immunostimulatory activities [51]. The qPCR data revealed that iSN40 did, indeed, initiate immune responses such as IL-1β induction, resulting in the abrogation of the RANKL-induced expression of osteoclastogenic genes (including *Nfatc1*, *Ctsk*, and *Dcstamp*), while iSN40 enhanced the macrophage transcription factor IRF-8 and a macrophage marker F4/80 (*Adgre1*), suggesting that iSN40 promotes the macrophage differentiation of RAW264.7 cells. CpG-ODNs have been reported to induce a chemokine, the C-C motif chemokine ligand 9 (CCL9), and its receptor, the C-C motif chemokine receptor 1 (CCR1), via TLR9 in RAW264.7 cells, resulting in macrophage activation [52]. Since TLR agonists with RANKL can arrest osteoclastogenesis in the early stages of differentiation [2], iSN40 would also direct RAW264.7 cells toward the macrophage lineage, and this may be part of the anti-osteoclastogenic mechanism.

In addition to iSN40, both iSN41 and CpG-2006 with CpG motifs inhibited osteoclastogenesis, suggesting that their anti-osteoclastogenic functions are mediated by a common mechanism. The structure simulation visually shows that the CpG motifs of iSN40 and iSN41 are stacked with the 5′-neighbor A, which may stabilize their CpG motifs and contribute to their recognition by TLR9 rather than other iSN40 homologs (iSN42–iSN47 have no anti-osteoclastogenic activities). Since both iSN40 and iSN41 induced IL-1β expression, they are considered to serve as immunostimulatory CpG-ODNs, similar to CpG-2006. These types of CpG-ODNs have been used as vaccine adjuvants and anti-cancer agents [9,53], and their safety has also been evaluated and confirmed. It can be proposed that iSN40, as a CpG-ODN, would be safe when used to suppress bone resorption.

In contrast, iSN40 and iSN40-GC were originally identified as osteoDNs that promote the mineralization and differentiation of osteoblasts independently of TLR9 at the optimal dose of 1–10 μM [21]. Although the pro-osteogenic mechanisms of iSN40 and iSN40-GC are still unknown, it has been hypothesized that they act as aptamers that specifically bind to the target molecule [21]. A molecular simulation revealed that the 3′ terminal AGCTT is structurally open and is highly accessible in iSN40 and iSN40-GC, but not in iSN41. These data suggest that the conformation of this region may be important for iSN40 and iSN41 to interact with their targets and promote osteogenesis.

iSN40 is expected to have a dual function, with pro-osteogenic and anti-osteoclastogenic effects, which simultaneously accelerate bone formation and suppress bone resorption in vivo. In this study, coculture experiments of MC3T3-E1 osteoblasts and RAW264.7 cells demonstrated that iSN40 can concurrently exert both the pro-osteogenic effect expressed as a reduced RANKL/OPG ratio and the anti-osteoclastogenic effect expressed as decreased resorption activity. A previous study reported that the osteoblast-specific knockout of the insulin receptor decreased the RANKL/OPG ratio and serum CTx level (a marker of bone resorption) in mice [54]. To clarify the dual role of iSN40 in the coculture system, the relationship between iSN40 and insulin signaling in osteoblasts needs to be further investigated. As romosozumab has demonstrated, a double action on both osteogenesis and osteoclastogenesis is beneficial for osteoporosis therapy [6,7]. iSN40 may be a promising nucleic acid drug for osteoporosis by modulating bone remodeling.

Note that the pro-osteogenic and anti-osteoclastogenic effects of iSN40 have been investigated in the murine cell lines, MC3T3-E1 and RAW264.7, respectively. The recognition of CpG-ODNs by TLR9 can be species-specific [24]. For example, CpG-1826 blocks osteoclastogenesis in mouse cells [23,26,27] but not in human cells [25]. Whether iSN40 can be recognized by human TLR9 has not been examined. The anti-osteoclastogenic effect of iSN40 needs to be investigated using human cells such as hPBMC. Similarly, the pro-osteogenic effect of iSN40 on human osteoblasts has not been established, and the detailed mechanism of action, including the direct target of iSN40, is still unknown. Another osteoDN, MT01, is also capable of directing osteogenesis but does not have CpG motifs [16,17,18,19]. While CpG-2006 is a CpG-ODN, it also interferes with osteoblastic differentiation in a TLR9-independent manner [20]. Since osteoblasts do not express TLR9 [21,55], the ODN’s functions on osteoblasts, regardless of their CpG motifs, are not thought to be based on TLR9. These ODNs would serve as nucleic acid aptamers that specifically interact with the target molecules (typically proteins) in a manner similar to antibodies [56]. It has been reported that bacterial genome-derived ODNs such as iSN40 can be aptamers and regulate cell fate [10]. Identification of the target of iSN40 as an aptamer in osteoblasts is essential for its future clinical application. In this study, iSN40 showed both pro-osteogenic and anti-osteoclastogenic effects in the coculture system in vitro. However, the intercellular signaling that regulates the balance of bone remodeling is more complex. The dual action of iSN40 in vivo needs to be confirmed by animal experiments such as an osteoporosis postmenopausal osteoporosis model.

**Figure 10:**
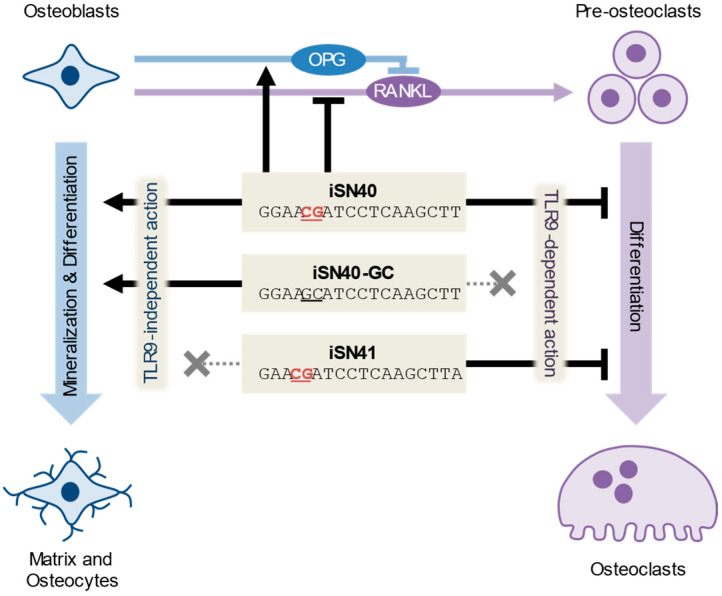
TLR9-independent pro-osteogenic and TLR9-dependent anti-osteoclastogenic effects of iSN40, iSN40-GC, and iSN41. CpG motifs are underlined.

Our previous and current studies proved that the pro-osteogenic and anti-osteoclastogenic effects of iSN40 can be separated. iSN40-GC without the CpG motif promoted the osteogenesis of MC3T3-E1 cells [21], but did not inhibit the osteoclastogenesis of RAW264.7 cells. In contrast, iSN41 with the CpG motif did not affect osteoblasts [21], while it did disrupt osteoclast formation (Figure 10). The optimal combination or proper use of these ODNs will achieve fine control of bone remodeling to balance bone formation and resorption, ultimately opening the door to the treatment of osteoporosis with nucleic acid drugs. Osteoporosis affects approximately 200 million people in the world and is associated with 8.9 million fractures per year [57]. The anti-sclerostin antibody, romosozumab [6,7], and anti-RANKL antibody, denosumab [58,59], are now widely used for osteoporosis therapy; however, antibody drugs are still expensive for a large number of patients worldwide. ODNs can be synthesized chemically, rapidly, and economically on a large scale [8,60]. iSN40, which exerts both pro-osteogenic and anti-osteoclastogenic effects, is a potent candidate for study and development as a nucleic acid drug for sustainable osteoporosis therapy.

## 5. Conclusions

This study demonstrated that iSN40, an 18-base CpG-ODN derived from the lactic acid bacterium genome, strongly inhibited RANKL-induced osteoclastogenesis through the recognition of its CpG motif by TLR9. A previous study reported that iSN40 promotes osteogenesis. The pro-osteogenic and anti-osteoclastogenic functions of iSN40 may be useful in maintaining the balance between bone formation and bone resorption. iSN40 is a potential nucleic acid drug for osteoporosis therapy by regulating bone remodeling.

## Figures and Tables

**Table 1:** ODN sequences.

ODN	Sequence (5′-3′)	Reference
iSN04	AGATTAGGGTGAGGGTGA	[10]
iSN40	GGAACGATCCTCAAGCTT	[10]
iSN40-GC	GGAAGCATCCTCAAGCTT	[21]
iSN41	GAACGATCCTCAAGCTTA	[10]
iSN42	AACGATCCTCAAGCTTAG	[10]
iSN43	ACGATCCTCAAGCTTAGG	[10]
iSN44	CGATCCTCAAGCTTAGGT	[10]
iSN45	GATCCTCAAGCTTAGGTC	[10]
iSN46	TCCTCAAGCTTAGGTCCG	[10]
iSN47	CCTCAAGCTTAGGTCCGC	[10]
BW001	TCGTCGGGTGCGACGTCGCAGGGGGG	[16]
BW006	TCGACGTTCGTCGTTCGTCGTTC	[16]
CpG-1585	GGGGTCAACGTTGAGGGGGG	[22]
CpG-1826	TCCATGACGTTCCTGACGTT	[23]
CpG-2006	TCGTCGTTTTGTCGTTTTGTCGTT	[24]
CpG-KSK13	TCGTCGTTTTCGTCGTCGTTTT	[25]
FC001	TCGGGGACGATCGTCGGGGAC	[16]
FC004	TCGCGTTCGATCGCGATCGACGGTA	[16]
MT01	ACCCCCTCTACCCCCTCTACCCCCTCT	[16]
YW001	TCGCGACGTTCGCCCGACGTTCGGTA	[16]
YW002	TCGCGAACGTTCGCCGCGTTCGAACGCGG	[16]

CpG motifs are underlined.

**Table 2:** Primer sequences for qPCR.

Gene	Primer Sequence (5′-3′)	Product	Reference
*Adgre1*	GAATCTTGGCCAAGAAGAGAC GAATTCTCCTTGTATATCATCAGC	157 bp	[31]
*Ctsk*	ACGGAGGCATTGACTCTGAAGATG GGAAGCACCAACGAGAGGAGAAAT	565 bp	[32]
*Dcstamp*	TCCTCCATGAACAAACAGTTCCAA AGACGTGGTTTAGGAATGCAGCTC	149 bp	[32]
*Il1b*	TTGACGGACCCCAAAAGATG CAGGACAGCCCAGGTCAAA	57 bp	[11]
*Irf8*	AGACCATGTTCCGTATCCCCT CACAGCGTAACCTCGTCTTCC	156 bp	[33]
*Nfatc1*	CAAGTCTCACCACAGGGCTCACTA GCGTGAGAGGTTCATTCTCCAAGT	119 bp	[32]
*Tlr1*	TCTCTGAAGGCTTTGTCGATACA GACAGAGCCTGTAAGCATATTCG	212 bp	[10]
*Tlr2*	TCTAAAGTCGATCCGCGACAT TACCCAGCTCGCTCACTACGT	344 bp	[10]
*Tlr3*	TTGTCTTCTGCACGAACCTG CGCAACGCAAGGATTTTATT	205 bp	[10]
*Tlr4*	CAAGAACATAGATCTGAGCTTCAACCC GCTGTCCAATAGGGAAGCTTTCTAGAG	278 bp	[10]
*Tlr5*	ACTGAATTCCTTAAGCGACGTA AGAAGATAAAGCCGTGCGAAA	401 bp	[10]
*Tlr6*	AACAGGATACGGAGCCTTGA CCAGGAAAGTCAGCTTCGTC	199 bp	[10]
*Tlr7*	TTCCGATACGATGAATATGCACG TGAGTTTGTCCAGAAGCCGTAAT	412 bp	[10]
*Tlr8*	GGCACAACTCCCTTGTGATT CATTTGGGTGCTGTTGTTTG	195 bp	[10]
*Tlr9*	TGCAATTGGCTGTTCCTGAA GGTGGTGGATACGGTTGGAG	100 bp	[10]
*Tlr11*	CCAGGACTGCACCTTTTGG GTGACACTGGTTGTACGCAAT	185 bp	[10]
*Tlr12*	AGAGCTGGCTGGTATGTTCC GTGTTCTTGTCAGGTCCAGAATC	161 bp	[10]
*Tlr13*	GGAGCGCCTTGATCTAACTAACA TCAGGTGGGTCAGAGAAACCA	80 bp	[10]
*Tnfsf11*	TCGGGTTCCCATAAAGTCAC TAGGTACGCTTCCCGATGTT	169 bp	This study
*Tnfrsf11b*	CGGAGACACAGCTCACAAGA GCTCGATTTGCAGGTCTTTC	243 bp	This study
*Ywhaz*	TTGATCCCCAATGCTTCGC CAGCAACCTCGGCCAAGTAA	88 bp	[34]

## Data Availability

The data presented in this study are available upon request to the corresponding author.
